# Cytotoxic effect of sodium hypochlorite (Lavanox 0.08%) and chlorhexidine gluconate (Irrisept 0.05%) on human osteoblasts

**DOI:** 10.1007/s00590-021-02907-3

**Published:** 2021-03-18

**Authors:** Sabrina Böhle, Eric Röhner, Timo Zippelius, Benjamin Jacob, Georg Matziolis, Sebastian Rohe

**Affiliations:** Orthopedic Department of the Waldkliniken Eisenberg, Orthopedic Professorship of the University Hospital Jena, Campus Eisenberg, Klosterlausnitzer Straße 81, 07607 Eisenberg, Germany

**Keywords:** Antiseptic solutions, Human osteoblasts, Cytotoxicity, Sodium hypochlorite, Chlorhexidine gluconate

## Abstract

**Purpose:**

Soft tissue, bone and joint infections are severe complications in orthopedic and traumatological surgery. Lavanox (0.08% NaOCl) and Irrisept (0.05% chlorhexidine gluconate, CHG) are industrially produced antiseptic solutions commonly used in infection treatment. Regarding this clinical indication, the microbicidal effect is often investigated, but toxicity to osteoblasts has rarely been examined. This is important to decide whether these solutions should be used in septic situations in which bone healing must take place. The hypothesis of the present study is that NaOCl and CHG are cytotoxic to osteoblasts even after a short exposure time.

**Methods:**

Human osteoblasts were isolated from donors with osteoarthritis during total knee and hip arthroplasty. Cells were cultivated and treated with both antiseptic solutions for 2, 5 and 10 min in different dilutions. Toxicity was quantified by counting cells, lactate dehydrogenase (LDH) expression, spectrophotometric quantification via XTT assay and FDA/PI fluorescence microscopy.

**Results:**

Analyzing viable cells after treatment with both antiseptics showed a significant decrease in viable cells through LDH expression test, XTT assay, fluorescence microscopy and light microscopy, depending on concentration. The time dependence showed a trend to more cell death at longer exposure times, without significance.

**Conclusion:**

Toxic effects on osteoblasts were shown after treatment with 0.08% NaOCl and 0.05% CHG after an exposure time of 2 min which also was concentration dependent. There was no difference in cytotoxicity between both antiseptics. In conclusion, these antiseptic solutions may be used with caution in situations requiring bone healing.

Trial registration number

Local ethics committee registration number: 5176–07/16

**Supplementary Information:**

The online version contains supplementary material available at 10.1007/s00590-021-02907-3.

## Introduction

Soft tissue, bone and joint infections are severe complications in orthopedic and traumatological surgery. Antiseptic solutions are used to reduce bacterial contamination in wounds. Industrially produced solutions in specific concentrations, e.g., sodium hypochlorite (NaOCl, Lavanox®, Serag-Wiessner GmbH, Naila, Germany) or chlorhexidine gluconate (CHG, Irrisept®, Irrimax Corporation, Lawrenceville, USA) are common in treatment of septic situations.

NaOCl is an effective agent against a wide range of microbes. It covers bacteria, mycobacteria, spores, viruses, algae and even protozoa [1]. In living organisms, hypochlorite is a useful biomolecule synthesized from hydrogen peroxide and chlorine ions catalyzed by the enzyme myeloperoxidase (MPO). It is secreted by activated phagocytes in zones of inflammation. HOCl is a key biological microbicidal agent, used as a natural defense owing to its great potency as a nucleophilic non-radical oxidant [2, 3].

Chlorhexidine is also an effective agent against microbes. In high concentrations, it damages the cell membrane, impairs DNA synthesis, and has a microbicidal effect. In lower concentrations, it acts by denaturation of cellular proteins with a bacteriostatic effect [4, 5].

Infection treatment in orthopedic and traumatological surgery must be performed frequently in situations requiring subsequent bone healing, e.g., fractures, osteotomies, arthrodesis, pseudarthrosis and bone grafts. The role of these topical antimicrobial agents is to limit bacterial proliferation without disturbing normal tissue or wound healing [6]. The microbicidal effect of both antiseptic solutions has been investigated extensively [4, 7, 8]. A cytotoxic time-dependent effect on fibroblasts (e.g., dermal and lung fibroblasts) in higher concentrations has also been described for both solutions [9–15].

To prevent tissue damage in our setting, the exposure time is limited to a few minutes, followed by irrigation with saline solution [16, 17], although most investigations about the bactericidal effect were performed using longer exposure times [4, 6–12, 18]. Nonetheless, there is a lack of knowledge about the effect of industrially solutions like Lavanox® or Irrisept® on osteoblasts, even after a shorter exposure time of just a few minutes.

The present study was therefore designed to investigate the effect of NaOCl (Lavanox® 0.08%) and chlorhexidine gluconate (CHG, Irrisept® 0.05%) on osteoblasts after different short exposure times and at different dilutions regarding an in vivo dilution due to local bleeding.

## Materials and methods

Tissue culture plasticware was obtained from BD (Germany). Culture medium, phosphate buffer saline (PBS), trypsin solution, fetal calf serum (FCS) and all other reagents were obtained from Invitrogen (Karlsruhe, Germany) and Sigma-Aldrich (Merck KGaA, Darmstadt, Germany).

Antiseptic solutions NaOCl (0.08% sodium hypochlorite, Lavanox®, Serag-Wiessner GmbH und Co. KG, Naila, Germany) and CHG (0.05% chlorhexidine gluconate, Irrisept®, Irrimax Corporation, Lawrenceville GA, USA) were purchased. Fresh dilutions were produced for each experiment. The products were used in their original concentration 1:1 and at 1:5 and 1:25 dilutions (CHG: 1:1 0.05%, 1:5 0.01%, 1:25 0.002%; NaOCl: 1:1 0.08%, 1:5 0.016%, 1:25 0.0032%).

### Osteoblast isolation, culture and treatment

Human bone tissue was obtained from seven donors who were suffering from knee or hip osteoarthritis according to a method previously described [19, 20]. Donors were included only if they did not present any signs of infection or tumor disease in clinical and blood examinations. After bone resection for total knee or total hip arthroplasty, the bone remnant was collected in a container under aseptic conditions. Within 2–5 h, bone fragments were separated from cartilage tissue and then minced manually into fragments sized 1–4 mm^3^. The bone fragments were then washed with PBS. Subsequently, they were suspended in osteogenic medium consisting of Dulbecco’s modified Eagle’s medium (DMEM) Ham’s F12 along with 10% FCS, 0.2% vitamin solution, 0.5% ß-glycerophosphate, 1% penicillin/streptomycin, and 0.01% dexamethasone, and finally cultured at 37 °C, 95% air and 5% CO_2_ establishing seven cell lines [19, 20]. After reaching subconfluency in a petri dish, cells were detached with 200 µl trypsin and transferred to a 75 cm^2^ and later to a 225 cm^2^ cell culture bottle.

Experiments were performed after osteoblast colonies had reached subconfluency again. Subsequently, osteoblasts were transferred and cultured on 24-well plates at a minimum density of 2.5 × 10^4^ cells per well with 500 µl of medium. Then, they were treated with either 0.08% NaOCl or 0.05% CHG and their dilutions (1:5, 1:25) as well as with PBS (for XTT, LDH and microscopy) and medium (for FDA/PI) (negative control) and 2% Triton X-100 (positive control) for 2, 5 and 10 min. Each investigation consists of a triplicate determination with at least six different cell lines. Dilutions were produced with aqua ad injectabilia (B. Braun Melsungen AG, Melsungen, Germany).

### Cell counting by light microscopy

After the described treatment above, human osteoblasts were washed with PBS and 200 µl trypsin releasing adherent cells. After this, a drop of the solution was placed into a counting chamber and counted by an automated cell counter (Cellometer Auto T4 Cell Counter, Nexcelom Bioscience, Lawrence, MA, USA).

### XTT ELISA detection of viable cells

The XTT assay was used for the spectrophotometric quantification of cell viability. This analysis is based on the cleavage of the tetrazolium salt XTT to form a formazan dye by metabolic active cells. This conversion only occurs in viable cells. An increase in XTT activity is directly proportional to an increasing number of living cells. After treatment described above, cells were washed with PBS and incubated with the XTT solution for 2 h. After this incubation period, a formazan solution was formed, which was spectrophotometrically quantified at 450–500 nm using an ELISA plate reader (FLUOstar OPTIMA, Microplate Reader, BMG LABTECH, Ortenberg, Germany).

### Detection of apoptosis and viability using fluorescence microscopy (FDA/PI)

Using fluorescence microscopy, the vital cells were stained with fluorescein diacetate (FDA) and the dead cells with propidium iodide (PI). FDA is taken up by cells that convert the non-fluorescent FDA into the green fluorescent metabolite fluorescein. The measured signal served as an indicator for viable cells, as the conversion was esterase dependent. In contrast, the nuclei staining dye PI cannot pass through a viable cell membrane. It reached the nucleus by passing through disordered areas of dead cell membranes and intercalated with the DNA double helix of the cell. After treatment described above and washing with PBS, cells were stained for 15 min with fluorescein diacetate and propidium iodide at a concentration of 20 µl per ml of medium. Documentation was undertaken immediately using fluorescence microscopy by counting viable and dead cells (microscope CKX 41, Olympus, Hamburg, Germany).

### Activity of lactate dehydrogenase (LDH)

LDH activity is a marker of advanced cell death. LDH exposure indicates the loss of membrane plasma integrity as a possible marker of increased cell necrosis [21, 22]. After treatment described above, LDH activity was measured immediately colorimetrically in the supernatant of the well plate at 490–492 nm in the ELISA plate reader (FLUOstar OPTIMA, Microplate Reader, BMG LABTECH, Ortenberg, Germany). Measuring the LDH activity after incubation with NaOCl was not reliable. In 2006, Kending et al*.* showed an inactivation of LDH using H_2_O_2_ and other oxidants in living cell lines. NaOCl is also a nucleophilic non-radical oxidant and could lead to LDH inactivation, while CHG does not act as an oxidant. Based on the fact that NaOCl falsifies the LDH measurement, such data were not available for NaOCl [23].

### Statistical analysis

Values were given as mean ± standard error of mean (SEM). To identify significant outliers, we used the Grubb’s test. The nonparametric Mann–Whitney U test was used as indicated in the legends. A *p* value of < 0.05 was considered to be significant.

## Results

Bone was collected from seven male subjects (two knees and five hips) during total joint arthroplasties. The average age was 62.1 years (range 51–77 years). Negative and positive control showed always a significant difference (*p* ≤ 0.001).

### Cell count by light microscopy

**Fig. 1:**
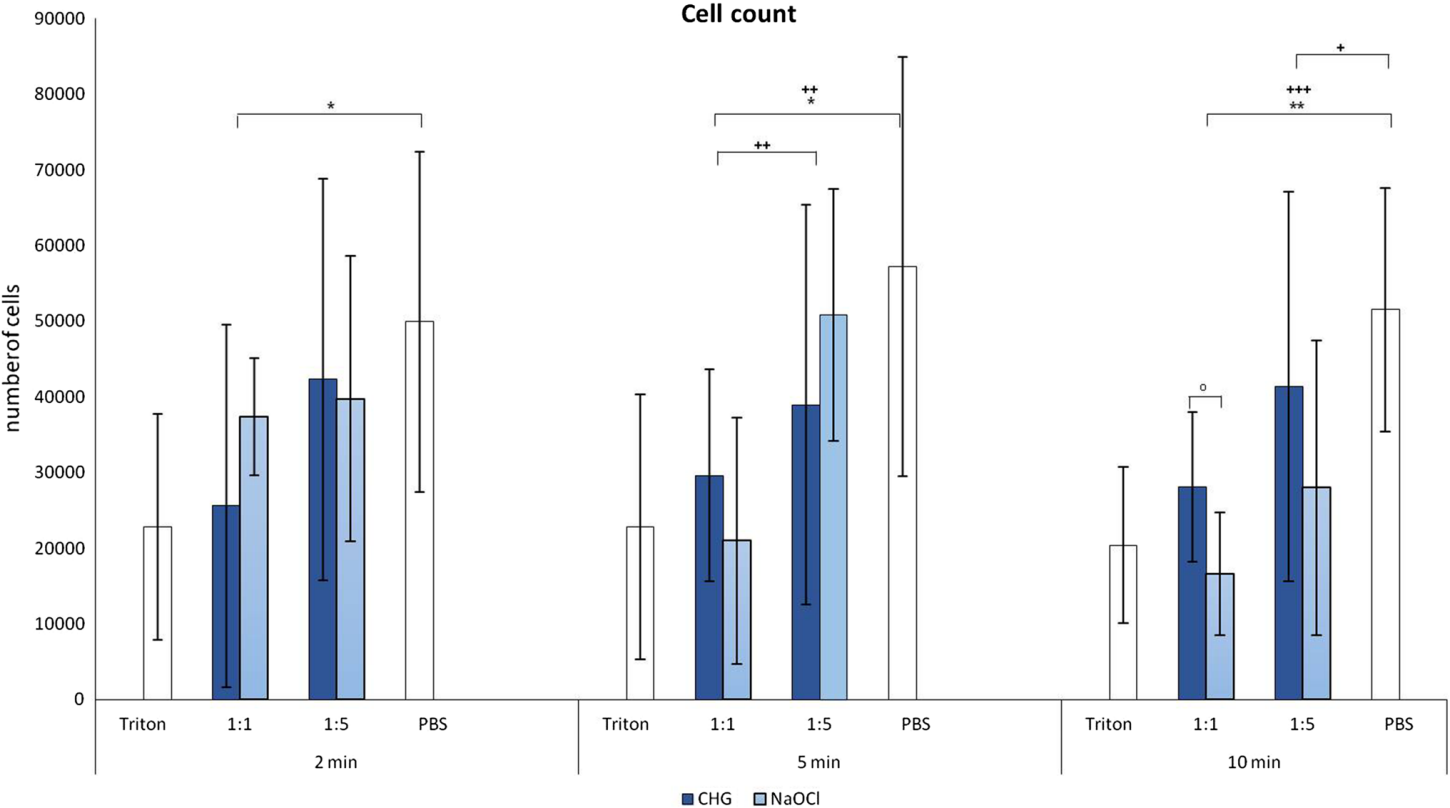
Comparison between the cell counts of osteocytes after the influence of CHG and NaOCl for 2, 5 and 10 min as well as their dilutions, positive and negative control. Values given as mean ± standard error of mean (SEM); CHG: * *p* ≤ 0.05; ** *p* ≤ 0.01; *** *p* ≤ 0.001; NaOCl ^+^ *p* ≤ 0.05; ^++^ *p* ≤ 0.01; ^+++^ *p* ≤ 0.001; CHG versus NaOCl ^o^ *p* ≤ 0.05

Using light microscopy for cell count, there was almost no cell death of osteoblasts when cultured with PBS (negative control) and a large number of dead cells after treatment with 2% Triton X-100 as known mediator of necrosis in human osteoblasts (Fig. 1). Non-diluted solutions of CHG at 2, 5 and 10 min and NaOCl at 5 and 10 min resulted in similar cytotoxicity. The cytotoxicity of non-diluted NaOCl after 2 min was lower than after 5 and 10 min, showing time dependence without reaching statistical significance. Both non-diluted solutions showed significantly fewer viable cells than PBS after 5 (*p* ≤ 0.03) and 10 min (*p* ≤ 0.004). CHG even shows a significant reduction of viable cells after 2 min (*p* ≤ 0.03). Diluted solutions of CHG and NaOCl showed a trend toward slightly more viable cells, without reaching statistical significance. After 10 min, non-diluted solutions of NaOCl showed a higher cytotoxicity than CHG. After 2 and 5 min of treatment, no significant difference was detectable (Fig. 1).

### XTT assay

**Fig. 2:**
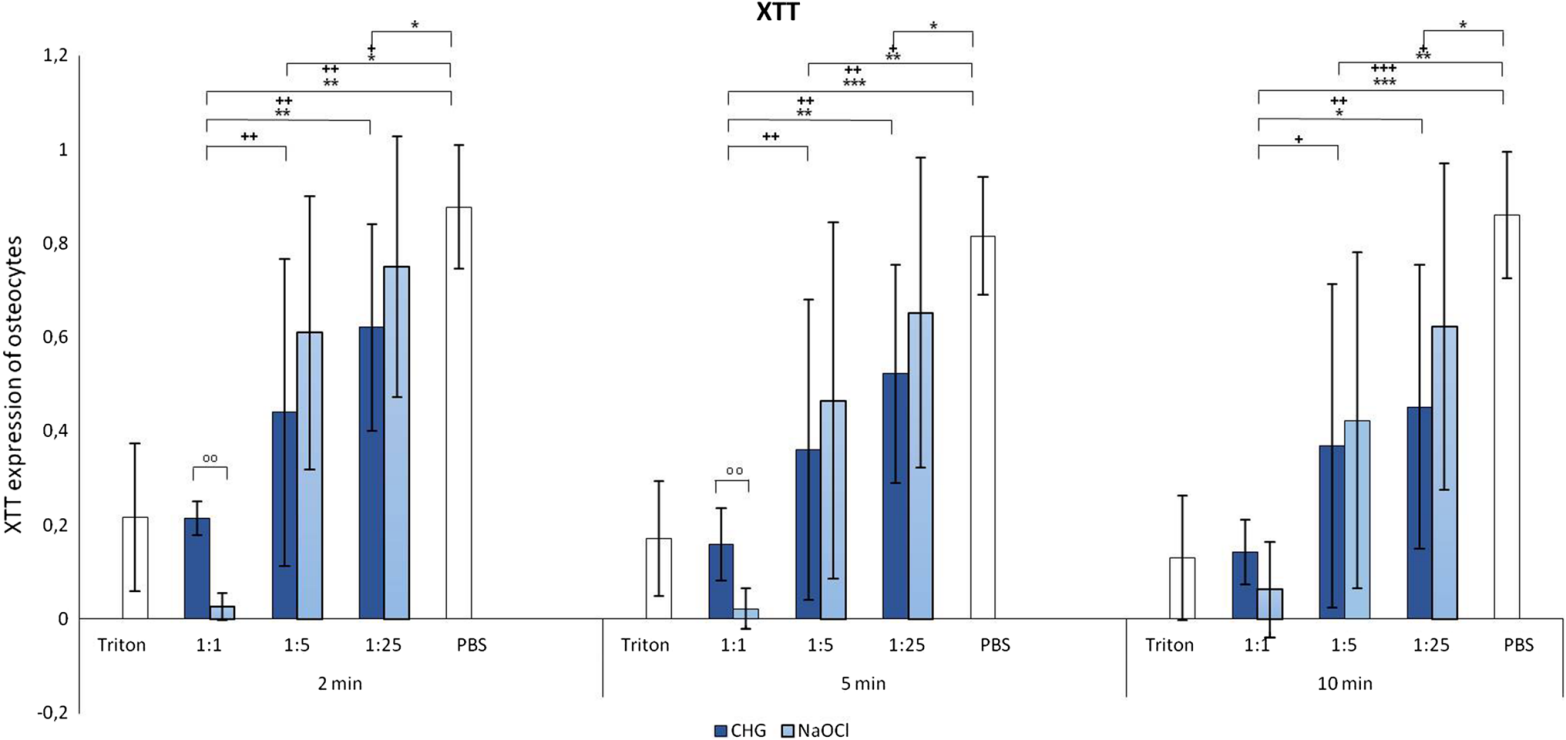
XTT assay for determination of cytotoxicity. Osteocytes were treated with 0.08% NaOCl and 0.05% CHG and their dilutions as well as two control groups with PBS (negative control) and 2% Triton X-100 (positive control) for 2, 5 and 10 min. Values given as mean ± standard error of mean (SEM); CHG: * *p* ≤ 0.05; ** *p* ≤ 0.01; *** *p* ≤ 0.001; NaOCl ^+^ *p* ≤ 0.05; ^++^ *p* ≤ 0.01; ^+++^ *p* ≤ 0.001; CHG versus NaOCl ^oo^ *p* ≤ 0.01

The XTT assay differentiated between viable and dead cells. There was almost no cell death with PBS (negative control), displayed by high XTT activity, and only a minimum XTT activity—indicating cell death—with Triton X-100 (positive control) (Fig. 2). Both antiseptic solutions showed a decrease in XTT activity depending on the exposure time, without reaching statistical significance. Both non-diluted and 1:5 diluted solutions showed significantly fewer viable cells than PBS at each time (*p* ≤ 0.002; *p* ≤ 0.035). At a 1:25 dilution only, CHG shows significant less XTT activity than PBS (*p* ≤ 0.035). We also detected significantly higher XTT activity at a 1:5 dilution of NaOCl and a 1:25 dilution of CHG, compared with non-diluted solutions, showing the concentration dependence (*CHG* after 2 min 1:1 vs. 1:25 p ≤ 0.004; after 5 min 1:1 vs. 1:25 *p* ≤ 0.026; after 10 min 1:1 vs. 1:25 *p* ≤ 0.026; *NaOCl* after 2 min 1:1 vs. 1:5 *p* ≤ 0.004; after 5 min 1:1 vs. 1:5 *p* ≤ 0.004; after 10 min 1:1 vs. 1:5 *p* ≤ 0.004). Except for non-diluted CHG and NaOCl, there was a trend toward more XTT activity—indicating cell viability—in the diluted NaOCl group compared with CHG over time and dilution. Against that, non-diluted NaOCl was more cytotoxic than non-diluted CHG after 2 and 5 min (*p* ≤ 0.02) (Fig. 2).

### *Fluorescence microscopy *via* FDA/PI staining*

**Fig. 3:**
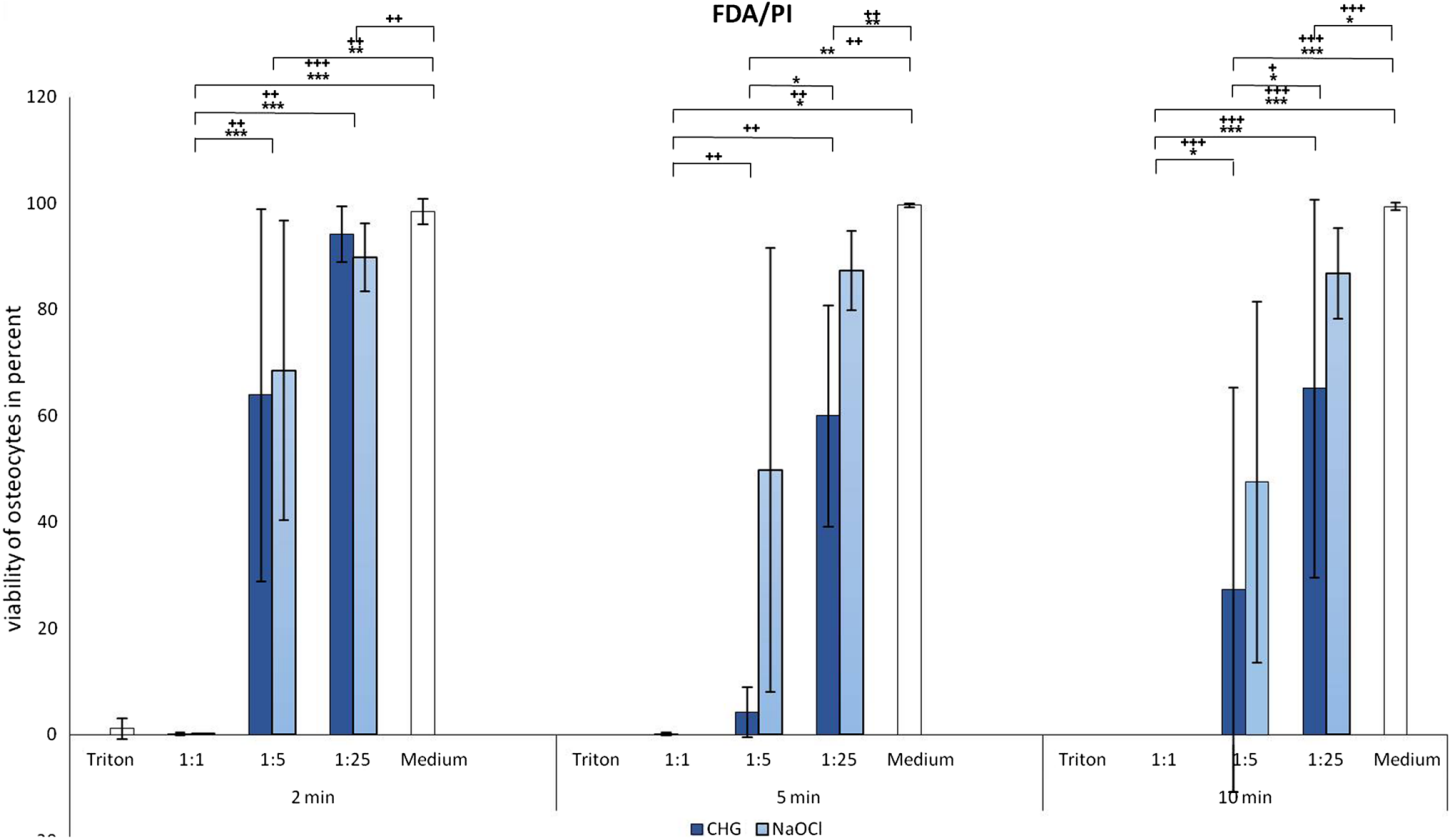
Detection of the vitality of osteocytes after treatment with CHG or NaOCl as well as with Triton X-100 and PBS for 2, 5 or 10 min by fluorescence microscopy with FDA/PI. Values given as mean ± standard error of mean (SEM); CHG: * *p* ≤ 0.05; ** *p* ≤ 0.01; *** *p* ≤ 0.001; NaOCl ^+^ *p* ≤ 0.05; ^++^ *p* ≤ 0.01; ^+++^ *p* ≤ 0.001

**Fig. 4:**
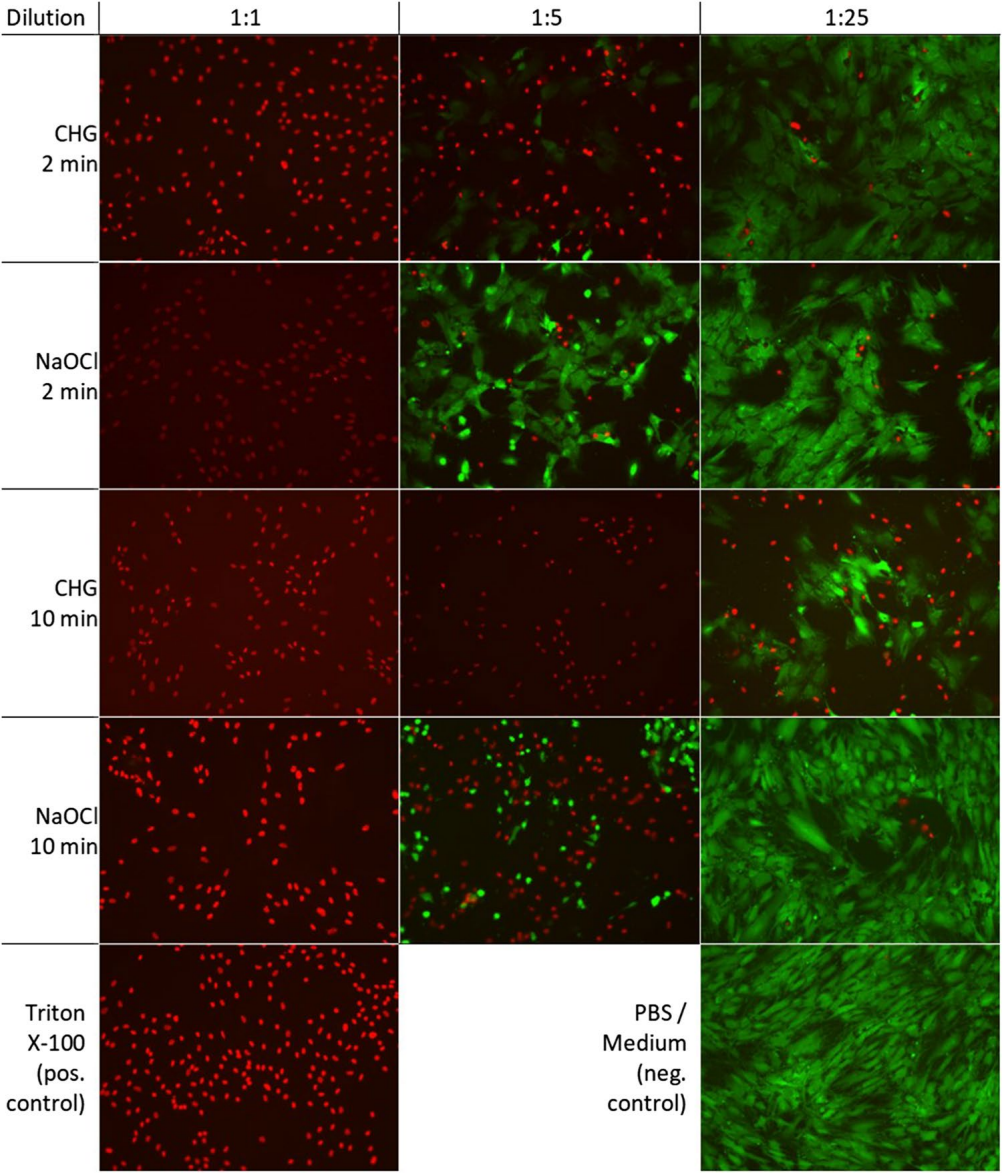
FDA/PI staining of viable osteoblasts after treatment with CHG and NaOCl for 2 and 10 min at different dilutions, as well as positive and negative control. Vital osteoblasts were FDA positive (green fluorescence) and PI negative. Necrotic cells were PI positive (red fluorescence) and FDA negative

Fluorescence microscopy was used to detect viable and dead cells (Figs. 3 and 4). There was almost no cell death with medium (negative control) and almost no viable cells with Triton X-100 (positive control). The non-diluted solutions of both antiseptics had low viable cell numbers like Triton X-100 after 2, 5 and 10 min. Furthermore, the 1:5 dilution of CHG showed a cytotoxic effect comparable to Triton X-100 after 5 and 10 min. In summary, both non-diluted solutions showed significant less viable cells than the medium control (*p* ≤ 0.003). Regarding CHG, a longer exposure time of 5 min led to fewer viable cells in comparable dilutions at 2 min (*p* ≤ 0.01), while the exposure time of NaOCl had no significant effect on cell viability. They showed a significantly higher number of viable cells depending on the dilution of the antiseptics (*CHG* after 2 min 1:1. vs. 1:5 *p* ≤ 0.001, after 5 min 1:1 vs. 1:25 *p* ≤ 0.029 and after 10 min 1:1 vs. 1:5 *p* ≤ 0.035; *NaOCl* after 2 min 1:1 vs. 1:5 *p* ≤ 0.002, after 5 min 1:1 vs. 1:5 *p* ≤ 0.008 and after 10 min 1:1 vs. 1:5 *p* ≤ 0.001). Comparing NaOCl and CHG, we showed slightly more viable cells in the diluted NaOCl than the CHG group after 5 and 10 min, without reaching significance (Fig. 3).

### LDH activity

**Fig. 5:**
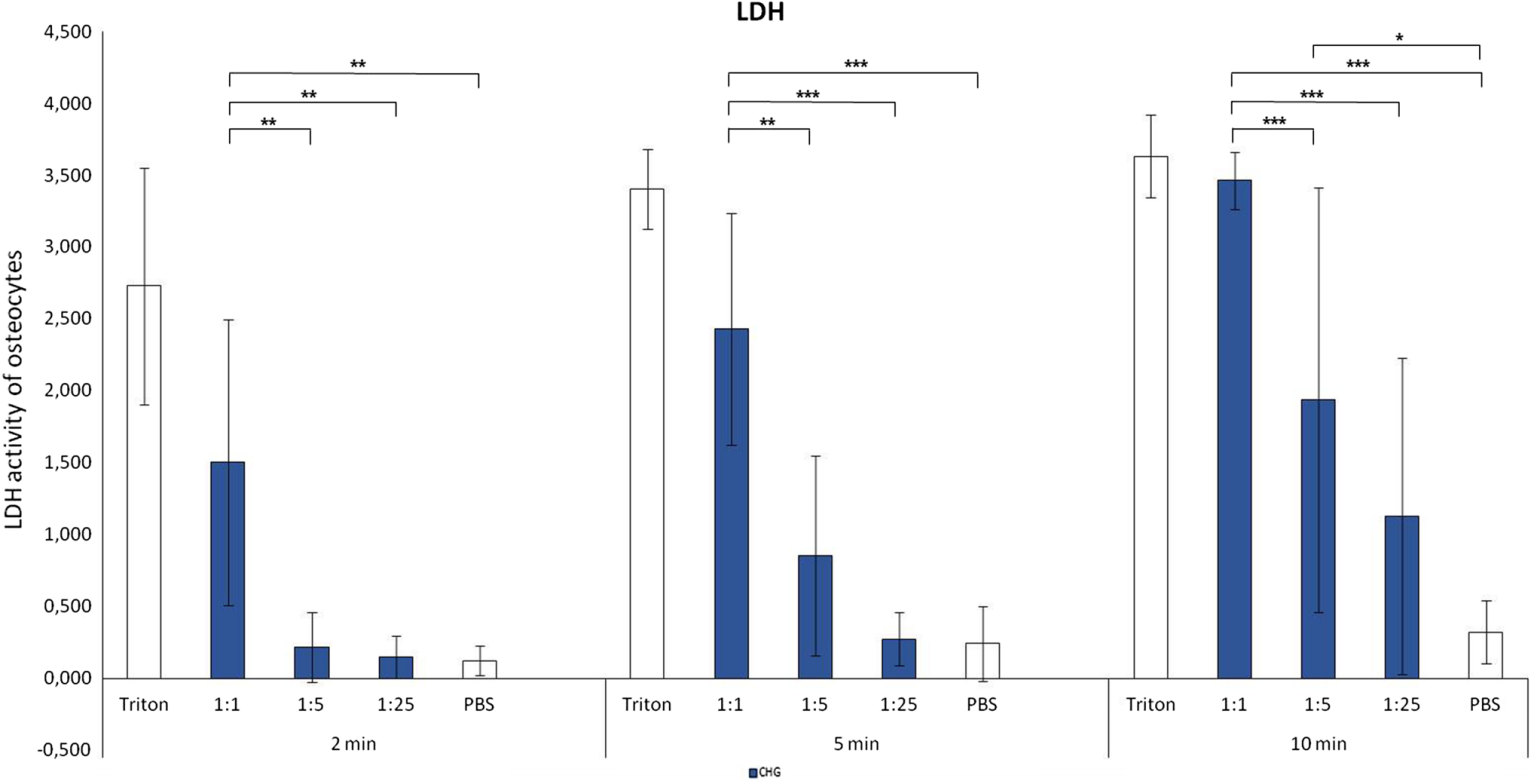
LDH activity as a marker of advanced cell death. Osteocytes were treated with 0.08% NaOCl and 0.05% CHG and their dilutions as well as two control groups with PBS (negative control) and 2% Triton X-100 (positive control) for 2, 5 and 10 min. Values given as mean ± standard error of mean (SEM); CHG: * *p* ≤ 0.05; ** *p* ≤ 0.01; *** *p* ≤ 0.001

LDH activity, indicating the onset of cell necrosis, was analyzed in the supernatant of each cell culture. There was almost no LDH release after incubation with PBS (negative control), but the highest concentration of LDH after incubation with Triton X-100 (positive control) after 2, 5 and 10 min (Fig. 4). After CHG treatment, the LDH activity increased with time of exposure. Non-diluted CHG showed significantly more cell necrosis than the negative control with PBS at each time (*p* ≤ 0.002). Furthermore, the LDH activity was concentration dependent, with higher LDH activity levels in less diluted CHG solutions at each time (*CHG* after 2 min 1:1 vs. 1:5 *p* ≤ 0.002; after 5 min 1:1 vs. 1:5 *p* ≤ 0.004; after 10 min 1:1 vs. 1:25 *p* ≤ 0.001). We did not obtain reliable results when measuring the LDH activity after incubation with NaOCl. In 2006, Kending et al*.* showed an inactivation of LDH using H_2_O_2_ and other oxidants in living cell lines [23]. NaOCl is also a nucleophilic non-radical oxidant and may lead to LDH inactivation, while CHG did not act as an oxidant. Consequently, a comparison between the two antiseptic solutions using the LDH activity test was not possible (Fig. 5).

## Discussion

The main result of the present study was that NaOCl and CHG in industrially produced concentrations were toxic to human osteoblasts even after a short exposure time of 2, 5 and 10 min. This effect was concentration and time dependent.

In septic orthopedic and traumatological surgery, antiseptic solutions are used after surgical debridement to further reduce the microbicidal contamination of the tissues. Both investigated solutions are commonly used for antiseptic wound rinsing, although there is a lack of evidence about cytotoxicity toward human osteoblasts after short exposure times. There are several situations in which bone healing should not be compromised (e.g., fractures, osteotomies, arthrodesis, pseudarthrosis, bone grafts), and the solutions investigated should only be used if strictly indicated.

In dentistry, NaOCl is often used for rinsing at high concentrations and has been investigated thoroughly. Kerbl et al*.* and Bi et al*.* reported compromised cancellous bone integrity in dog and calf femur in an endodontic setting at high NaOCl concentrations (5.25% and 10%) [8, 9]. These data are supported by Goffin et al*.* who reported skin damage with comparably highly concentrated NaOCl (4%) [15]. Lineweaver et al*.* reported total destruction of fibroblasts after 15-min exposure with less concentrated NaOCl (0.5%) [6]. These effects seem to be concentration dependent according to the data of Kozol et al*.,* who investigated neutrophil, fibroblast and endothelial cell viability after treatment with 0.025–0.0025% NaOCl for an exposure time of 30 min and reported cytoplasmic vacuolation, swollen mitochondria and dilated endoplasmic reticulum in fibroblasts and endothelial cells, but no cell deaths [14]. Sawada et al*.* showed a decrease in viable cells after 10-min rinsing with 0.25% NaOCl [11]. Vouzara et al*.* reported a cytotoxic effect of NaOCl on MRC5 cells (human lung fibroblasts) [12] and Hidalgo et al*.* on dermal fibroblasts, depending on concentration and exposure time [10]. Hidalgo et al*.* also investigated cytotoxicity of 0.00005 to 0.1% NaOCl to dermal fibroblasts after an exposure time of 2 to 24 h. Beginning cytotoxicity was described at a concentration of 0.0075% NaOCl after 8 h, increasing with concentrations higher than 0.025%, and leading to almost total cytotoxicity at 0.05% NaOCl [10]. Hidalgo et al*.* also showed a partial bactericidal effect of NaOCl at concentrations varying from 0.25 to 0.025%. NaOCl concentrations below 0.005% were totally devoid of bactericidal effects; for gram-negative bacteria, the cutoff is even 0.025% [10]. But there is still a lack of knowledge about the effect on human osteoblasts of less concentrated NaOCl after a short exposure time for intraoperative rinsing. In our study, we used 0.08% NaOCl and exposure times from 2 to 10 min and confirmed the reported cytotoxicity to osteoblasts, even after an essential shorter exposure time.

Hidalgo et al. also investigated the concentration-dependent cytotoxicity of CHG (0.0005 to 0.025%) to dermal fibroblasts after an exposure time of 3 to 24 h. They showed almost total cell death after 3 h for concentrations ≥ 0.005% CHG [5]. Vouzura et al*.* reported an even greater cytotoxic effect of CHG than that of NaOCl [12]. Sawada et al*.* showed a cytotoxic effect of CHG on the mandibular bone of pigs in vitro [4]. Van Meurs et al*.* reported a high cytotoxicity of CHG at a concentration required for a sufficient bactericidal effect [7]. They showed a concentration-dependent cytotoxicity of CHG on human fibroblasts after 2-min exposure, a minimal concentration for bactericidal effects of 0.078%, and a measurable cytotoxic effect beginning at 0.002% CHG [7]. Also, Liu et al*.* reported a concentration-dependent cell necrosis with survival rates of human myoblasts, fibroblasts and osteoblasts lower than 6% at concentrations ≥ 0.02% CHG [13]. In our study, we used a 0.05% CHG solution and confirmed the reported cytotoxicity for human osteoblasts for an exposure time between 2 and 10 min.

Comparing CHG to NaOCl, Sawada et al*.* reported a higher cytotoxic effect of NaOCl than CHG after investigating antiseptic solution in dentistry bone samples. They showed higher cell viability after rinsing with 0.2% CHG than 0.25% NaOCl for different exposure times [4, 11]. On the other hand, Vouzara et al*.* reported a higher cytotoxic effect of CHG than NaOCl on MRC5 cell samples at different concentrations of the solutions after 6 and 24 h [12]. In our study, there was no significant consistent difference in cytotoxicity between both solutions.

In the present work, direct effects of 0.08% NaOCl and 0.05% chlorhexidine gluconate on human osteoblasts were examined. We were able to demonstrate cell damage on the basis of cell necrosis [21, 22] and a reduction of viable cells after treatment with both antiseptic solutions, depending on exposure time and dilution.

This study has several limitations. The use of human osteoblasts from bone of patients with knee osteoarthritis yields bone tissue that is not fully intact, compared with the tissue in healthy people without osteoarthritis. The results of our study do not represent the in vivo situation, as in vitro results show higher cell toxicity than in vivo investigations based on direct osteoblast treatment without contact to blood and protecting serum components. In order to this, we tried to simulate the in vivo dilution by an artificial 1:5 dilution. Additionally, antiseptic solutions in vitro do not have to pass different barriers like intercellular matrix.

## Conclusion

In summary, extensive toxic effects of 0.08% NaOCl and 0.05% CHG in industrially produced solutions on human osteoblasts were present even after a short incubation time of 2 min and after a dilution of 1:5 regarding in vivo dilutions due to bleeding. Comparing both solutions, there was no significant difference in their cytotoxicity to human osteoblasts consistently. Based on our results, both antiseptic solutions may be used with caution in situations requiring bone healing (e.g., fractures, osteotomies, arthrodesis, pseudarthrosis, and bone grafts). Therefore, we recommend a short exposure time less than 5 min and a wound rinsing with sterile saline solution after using them. We favor the use of Lavanox® because of a wider  microbicidal range against different types of microbes even with adherent biomembranes [1, 6, 24], but with subsequent lavage within 2–3 min contrary to manufacturer’s recommendation.

## Supplementary Information

Below is the link to the electronic supplementary material.

Supplementary file1 (PDF 188 KB)

## Data Availability

All data are available by contacting the corresponding author.
