# Supplementary material for: Cytotoxic effect of sodium hypochlorite (Lavanox 0.08%) and chlorhexidine gluconate (Irrisept 0.05%) on human osteoblasts
Source: Eur J Orthop Surg Traumatol. 2021 Mar 18;32(1):81–9. doi: 10.1007/s00590-021-02907-3 (PMC8741695; doi:10.1007/s00590-021-02907-3)
Supplement: Supplementary file 1 — Supplementary file1 (PDF 188 KB) [file 590_2021_2907_MOESM1_ESM.pdf]

Supplemental Table 1: descriptive statistics

			2 min					5 min					10 min				
			**Triton**	**1:1**	**1:5**	**1:25**	**PBS**	**Triton**	**1:1**	**1:5**	**1:25**	**PBS**	**Triton**	**1:1**	**1:5**	**1:25**	**PBS**
**Cell Count**	**CHG**	mean	22813,97	25607,86	42301,90	40991,67	49942,86	22815,67	29626,43	38974,76	40870,48	57196,67	20395,13	28083,57	41417,14	37201,11	51526,90
		SEM	14889,02	23997,16	26549,85	29297,69	22472,12	17490,36	13962,26	26408,72	15543,52	27740,36	10329,05	9930,97	25739,63	26235,98	16106,84
	**NaOCl**	mean	22813,97	37377,14	39739,52	42335,24	49942,86	22815,67	21025,48	50879,52	49326,19	57196,67	20395,13	16603,61	28003,33	41177,14	51526,90
		SEM	14889,02	7782,38	18870,15	22767,37	22472,12	17490,36	16286,30	16666,82	26737,23	27740,36	10329,05	8156,38	19482,83	18964,78	16106,84
**XTT**	**CHG**	mean	0,22	0,21	0,44	0,62	0,88	0,17	0,16	0,36	0,52	0,82	0,13	0,14	0,37	0,45	0,86
		SEM	0,16	0,04	0,33	0,22	0,13	0,12	0,08	0,32	0,23	0,13	0,13	0,07	0,34	0,30	0,13
	**NaOCl**	mean	0,22	0,03	0,61	0,75	0,88	0,17	0,02	0,47	0,65	0,82	0,13	0,06	0,42	0,62	0,86
		SEM	0,16	0,03	0,29	0,28	0,13	0,12	0,04	0,38	0,33	0,13	0,13	0,10	0,36	0,35	0,13
**FDA/PI**	**CHG**	mean	1,08	0,15	63,87	94,16	98,43	0,00	0,11	4,14	59,99	99,60	0,00	0,00	27,23	65,11	99,35
		SEM	2,01	0,29	35,01	5,20	2,44	0,00	0,22	4,68	20,82	0,35	0,00	0,00	38,02	35,57	0,72
	**NaOCl**	mean	1,08	0,09	68,54	89,84	98,43	0,00	0,00	49,74	87,30	99,60	0,00	0,00	47,49	86,78	99,35
		SEM	2,01	0,15	28,15	6,36	2,44	0,00	0,00	41,77	7,52	0,35	0,00	0,00	33,91	8,54	0,72
**LDH**	**CHG**	mean	2,73	1,50	0,22	0,15	0,12	3,41	2,43	0,85	0,27	0,24	3,63	3,46	1,94	1,13	0,32
		SEM	0,82	0,99	0,24	0,15	0,10	0,28	0,81	0,70	0,18	0,26	0,29	0,20	1,48	1,10	0,22
